# Severe maternal morbidity due to respiratory disease and impact of 2009 H1N1 influenza A pandemic in Brazil: results from a national multicenter cross-sectional study

**DOI:** 10.1186/s12879-016-1525-z

**Published:** 2016-05-21

**Authors:** L. C. Pfitscher, J. G. Cecatti, R. C. Pacagnella, S. M. Haddad, M. A. Parpinelli, J. P. Souza, S. M. Quintana, F. G. Surita, M. H. Sousa, M. L. Costa, Brazilian Network for Surveillance of Severe Maternal Morbidity Group, Joao L. Pinto Silva, Rodrigo S. Camargo, Vilma Zotareli, Lúcio T. Gurgel, Eliana M Amaral, Lale Say, Robert C. Pattinson, Marilza V. Rudge, Iracema M. Calderon, Maria V. Bahamondes, Danielly S. Santana, Simone P. Gonçalves, Olímpio B. Moraes Filho, Simone A. Carvalho, Francisco E. Feitosa, George N. Chaves, Ione R. Brum, Gloria C. Saint’Ynes, Carlos A. Menezes, Patricia N. Santos, Everardo M. Guanabara, Elson J. Almeida, Joaquim L. Moreira, Maria R. Sousa, Frederico A. Peret, Liv B. Paula, Luiza E. Schmaltz, Cleire Pessoni, Leila Katz, Adriana Bione, Antonio C. Barbosa Lima, Edilberto A. Rocha Filho, Melania M. Amorim, Debora Leite, Ivelyne Radaci, Marilia G. Martins, Frederico Barroso, Fernando C. Oliveira, Denis J. Nascimento, Cláudio S. Paiva, Moises D. Lima, Djacyr M. Freire, Roger D. Rohloff, Simone M. Rodrigues, Sergio M. Costa, Adriana G. Luz, Daniela Guimaraes, Gustavo Lobato, Marcos Nakamura-Pereira, Eduardo Cordioli, Alessandra Peterossi, Cynthia D. Perez, Jose C. Peraçoli, Roberto A. Costa, Nelson L. Maia Filho, Jacinta P. Matias, Elaine C. Moises, Fátima A. Lotufo, Luiz E. Carvalho, Carla B. Andreucci, Elvira A. Zanette, Márcia M. Aquino, Maria H. Ohnuma, Rosiane Mattar, Felipe F. Campanharo

**Affiliations:** Department of Obstetrics and Gynecology, University of Campinas (UNICAMP), School of Medicine, Alexander Fleming, 101, 13083-881 Campinas, São Paulo Brazil; Department of Obstetrics and Gynecology, University of São Paulo School of Medicine in Ribeirão Preto (USPRP), São Paulo, Brazil

**Keywords:** Maternal morbidity, Maternal mortality, Maternal near miss, H1N1, Respiratory disease

## Abstract

**Background:**

The aim of this study was to assess the burden of respiratory disease, considering the influenza A pandemic season (H1N1pdm09), within the Brazilian Network for Surveillance of Severe Maternal Morbidity, and factors associated with worse maternal outcome.

**Methods:**

A multicenter cross-sectional study, involving 27 referral maternity hospitals in five Brazilian regions. Cases were identified in a prospective surveillance by using the WHO standardized criteria for potentially life-threatening conditions (PLTC) and maternal near miss (MNM). Women with severe complications from respiratory disease identified as suspected or confirmed cases of H1N1 influenza or respiratory failure were compared to those with other causes of severe morbidity. A review of suspected H1N1 influenza cases classified women as non-tested, tested positive and tested negative, comparing their outcomes. Factors associated with severe maternal outcome (SMO = MNM + MD) were assessed in both groups, in comparison to PLTC, using PR and 95 % CI adjusted for design effect of cluster sampling.

**Results:**

Among 9555 cases of severe maternal morbidity, 485 (5 %) had respiratory disease. Respiratory disease occurred in one-quarter of MNM cases and two-thirds of MD. H1N1 virus was suspected in 206 cases with respiratory illness. Around 60 % of these women were tested, yielding 49 confirmed cases. Confirmed H1N1 influenza cases had worse adverse outcomes (MNM:MD ratio < 1 (0.9:1), compared to 12:1 in cases due to other causes), and a mortality index > 50 %, in comparison to 7.4 % in other causes of severe maternal morbidity. Delay in medical care was associated with SMO in all cases considered, with a two-fold increased risk among respiratory disease patients. Perinatal outcome was worse in cases complicated by respiratory disease, with increased prematurity, stillbirth, low birth weight and Apgar score < 7.

**Conclusions:**

Respiratory disease, especially considering the influenza season, is a very severe cause of maternal near miss and death. Increased awareness about this condition, preventive vaccination during pregnancy, early diagnosis and treatment are required to improve maternal health.

## Background

Improvement in maternal health aiming a reduction in maternal mortality is a priority worldwide, in an attempt to accomplish the established millennium development goals set for 2015 [1–3]. However, the expected reduction in maternal mortality is still far from ideal and varies widely across regions [4, 5]. Most recently, to better comprehend the burden of disease on female health and complement mortality inquiries, an alternative approach has been to study maternal morbidity. Maternal morbidity can have an impact on both low-income and high-income settings.

In 2009, the World Health Organization (WHO) standardized the definitions of potentially life-threatening conditions (PLTC) and maternal near miss (MNM) [6]. PLTC is defined by the number of maternal complications, including hemorrhagic (e.g., abruption placenta, ruptured uterus, atony and others), hypertensive disorders (e.g., severe preeclampsia, eclampsia, HELLP syndrome), management indicators of severity (e.g., blood transfusion, intubation, intensive care unit admission) and other complications (e.g., pulmonary edema, cardiac disease and sepsis). Maternal near miss (MNM) is any situation in which a woman survives a very severe complication with proven organ dysfunction, during pregnancy or puerperium (42 days after childbirth), with at least one of the following criteria: clinical (e.g., shock or clotting disorder), laboratory (lactate > 5, PaO2/FiO2 < 200 mmHg) or management (hysterectomy due to infection or hemorrhage and blood transfusion ≥ 5 units of packed red blood cells). Severe Maternal Outcome (SMO) accounts for cases of MNM plus Maternal Deaths (MD) [6].

Recently, the concept of “obstetric transition” was incorporated into the study of maternal morbidity and mortality. The concept illustrates a global trend in which a pattern of high maternal mortality rates with predominantly direct obstetric causes (e.g., hemorrhage, preeclampsia or uterine infection) has been replaced by lower maternal mortality rates with an increasing proportion of indirect causes (preexisting disorders or those aggravated by pregnancy, such as cardiac disease, kidney disease or infection due to urinary or pulmonary foci), institutionalization and medicalization of childbirth and increased rate of obstetric interventions [7]. Obstetric transition is important to help understand the occurrence of severe maternal morbidity and provide patients with the appropriate treatment in different settings.

Among the indirect causes of maternal morbidity and mortality, respiratory disease plays a significant role, either due to the presence of severe infection or complications of the underlying conditions, such as asthma and heart disease. Physiological and anatomical changes that occur during pregnancy to provide accommodation for the growing uterus, can affect the known clinical presentation of respiratory signs and symptoms. Adequate diagnosis and treatment of respiratory disease may be delayed [8, 9]. In addition, it is recognized that pregnancy may increase the risk of severe influenza-associated complications [10, 11].

It became clear throughout the 2009 H1N1 influenza pandemic [termed A(H1N1)pdm09] worldwide [12–17] that pregnant women were a highly vulnerable group. From July 2009 to January 2, 2010, 44,544 cases of the disease and 2051 deaths were reported in Brazil, [18]. However, the total number of cases and deaths were likely much higher than the notified number.

We proposed a novel approach to analyzing the burden of 2009 H1N1 influenza virus infection and other respiratory disease among patients with severe maternal morbidity. Cases complicated by severe respiratory disease were compared to cases with morbid conditions due to other causes (such as hemorrhage and hypertension). In addition, factors possibly associated with a higher risk of SMO were evaluated by using the WHO standardized definitions of morbidity in 27 referral maternity hospitals.

## Methods

This study is a secondary analysis of the Brazilian Network for Surveillance of Severe Maternal Morbidity including 27 referral maternity hospitals in Brazil. The study evaluated severe maternal morbidity cases, from a prospective surveillance, according to the 2009 WHO newly publicized criteria for these conditions [6].

The methodological details of the original study have already been published elsewhere [19, 20]. Briefly, this multicenter study included 27 referral maternity hospitals distributed among the five Brazilian geographical regions. From July 2009 to June 2010, all women admitted to participating centers, who were identified as having any life-threatening condition, near miss or maternal death, according to the WHO definition, were included in the study. Data collection, by the study team, was acquired through medical chart review after hospital discharge or death of the patient. If any doubt on diagnosis considered, the treating doctors were further contacted for clarifications. Information was entered into the OpenClinica® electronic platform (version 2.5.5 - Waltham, MA, USA) through a structured form completed by the local coordinator from each participating center.

This was not a population based study, however, there was a concern to reduce the impact of nonrandom sampling and an effort to consider representativeness of the national territory (with health facilities from all five macro-regions of the country) and of facilities from public and private sectors, university and non-university hospitals. All selected hospitals had to provide information concerning their characteristics, including location, complexity of level of care, population covered, number of maternity beds and availability of resources for severe cases.

Quality control was carried out during various phases of the study. Initially, training was provided to the entire team participating in the study, using a detailed operations manual, with the definition of each variable. Meetings were held between the local research team and the coordinating team of the study to standardize data. Case review was conducted by the local investigator. Subsequently, the coordinating team of the study performed random reviews of manual and electronic forms for data consistency in visits to monitor the centers’ performance. Periodically, review of the electronic system was carried out to check for data inconsistency, along with systematic case review. Some reported conditions were delay or substandard care, which had been previously reported [21]. Reasons for the delay in treatment were the woman or family member (including delay in identifying the condition, seeking care and refusing to accept treatment), health service (difficulties in obtaining equipment or medical supplies) or health professional (delays in identifying the correct diagnosis and providing appropriate patient treatment). Sample size was determined by the prevalence of about 8 maternal near miss cases per 1000 births and a maternal mortality ratio of 140/100,000 live-born infants (95 % confidence interval). It was predicted that 75,000 births [19] needed to be monitored.

For the present analysis, we considered severe respiratory disease as a suspected or confirmed case of influenza or acute respiratory failure, defined as incapacity of the respiratory system to promote adequate gas exchange, with arterial blood gas parameters: PaO2 < 60 mmHg or peripheral saturation < 90 %, associated or not with PaCO2 > 50 mmHg. Clinical parameters such as tachypnea (respiratory rate-RR > 20) or bradypnea (RR <6), use of accessory respiratory muscles, nasal flaring, associated with torpor or agitation were also considered. For suspected or confirmed cases of A(H1N1)pdm09, the definition of cases considered only those with severe morbidity, including acute respiratory insufficiency, sepsis, intensive care admission, intubation and others. Cases of H1N1 without severe complications were not included. A review of all H1N1 Influenza cases was necessary to confirm whether laboratory tests had been performed and to obtain the results of these tests, since data in the original study had not been collected in detail. Case review was requested from each local center and new data were distributed into three groups: non-tested, positive and negative cases for H1N1 influenza virus.

Initially, the prevalence of PLTC, MNM and MD was calculated per group, as well as the respective health indicators related to maternal morbidity and mortality: maternal near miss ratio, severe maternal outcome ratio, mortality index and maternal mortality ratio, according to the WHO definition [6]. To evaluate the progression of severe maternal morbidity in cases complicated by respiratory disease throughout the study, maternal outcomes (PLTC, MNM and MD) were measured for each month studied. The risk of SMO associated with procedures used to manage the severity of conditions was estimated for the group with severe respiratory disease and other causes of severe maternal morbidity, using Prevalence Ratios plus their respective 95 % CI adjusted for the design effect of cluster sampling.

Subsequently, we performed an analysis considering the total number of cases with severe respiratory disease versus cases with other causes of severe maternal morbidity. In each group, PLTC (less severe cases) and Severe Maternal Outcome (SMO: MNM + MD) cases were compared to evaluate the factors potentially associated with more severe disease, including delay in obstetric care, also using the Prevalence Ratios plus their respective 95 % CI adjusted for the design effect of cluster sampling. The prevalence of sociodemographic, obstetric and perinatal factors were evaluated between the two groups using Chi-square tests. Values statistically significant were considered those with a p-value under 0.05. The statistical procedures for analysis were performed with SPSS and Stata.

## Results

**Fig. 1:**
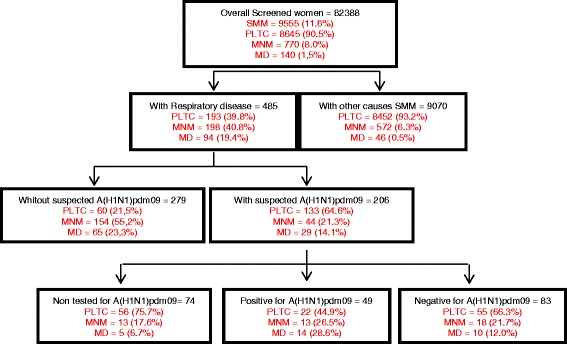
Flow chart of women with severe maternal morbidity (SMM = PLTC + MNM + MD) due to severe respiratory disease or other causes of morbidity and suspected influenza A(H1N1)pdm09 as positive, negative or not tested, considering the final maternal outcome in potentially life-threatening condition (PLTC), maternal near miss (MNM), or maternal death (MD)

During the 12-month study period, 82,388 women were screened. Of these, 9555 had criteria for severe maternal morbidity. Among these 9555 women, only 485 (5 %) had severe respiratory disease. However, in this group with respiratory illness, symptom severity progressed more rapidly, if compared to other causes of severe morbidity (Fig. 1), such as bleeding or hypertensive disorders, and may be 40 times more lethal.

Among the total number of women with respiratory disease, patients with suspected H1N1 influenza A virus infection had more severe disease (55.2 % MNM and 23.3 % MD) than those without suspected H1N1 influenza A virus (prevalence of MNM: 21.3 %, MD: 14.1 %) (Fig. 1). About 60 % of cases of suspected H1N1influenza A were tested. Women who tested positive (49 cases) for H1N1 had more severe disease, with a higher prevalence of SMO.

**Fig. 2:**
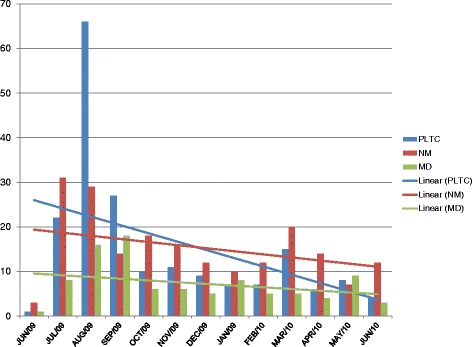
Distribution of cases with severe respiratory disease, according to progression of severity during the study period

Figure 2 shows the distribution of cases with severe respiratory disease, according to progression of severity during the study period, based on date of admission in participating centers. There was a higher incidence of cases in the first months considered, especially July, August and September 2009. National guidelines and availability of vaccination during pregnancy were instituted in March/2010.

Considering health indicators, disease was more severe among cases tested and positive for H1N1 (Table 1). Mortality rate was higher than 50 % among positive cases for A(H1N1)pdm09. The death rate was about 36 % in cases testing negative for H1N1 and 27.5 % in non-tested cases. In contrast, the mortality rate was only 7.4 % in morbid disorders due to other causes. The maternal near miss to mortality ratio was 0.93:1, 1.80:1 and 2.39:1, among positive, negative and non-tested groups for A(H1N1)pdm09, respectively, compared to a value of 12.43:1 for other causes of severe maternal morbidity.

**Table 1:** Women with severe respiratory disease: cases non-tested for influenza A(H1N1)pdm09, influenza A(H1N1)pdm09 positive, influenza A(H1N1)pdm09 negative and other causes of morbidity according to severity of outcome (PLTC, MNM, MD) and their correspondent health indicators

Conditions	PLTC	MNM	MD	Total	Health indicators				
					MNMR/ 1000LB	SMOR/ 1000LB	MNM:MD ratio	Mortality index %	MMR/ 100000LB
Influenza A(H1N1)pdm09 positive	22	13	14	49	0.16	0.33	0.93:1	51.8	17.0
Influenza A(H1N1)pdm09 negative	55	18	10	83	0.22	0.34	1.80:1	35.7	12.2
Non-tested for influenza A(H1N1)pdm09	116	167	70	353^a^	2.03	2.89	2.39:1	29.5	85.2
Total Respiratory disease	193	198	94	485	2.41	3.55	2.11:1	32.2	114.4
Other causes	8452	572	46	9070	6.96	7.52	12.43:1	7.4	56.0

LB: 82.144

LB: live births; PLTC: potentially life-threatening condition; MNM: maternal near miss; MD: maternal death; MNMR: maternal near miss ratio; SMOR: severe maternal outcome ratio = (MNM + MD)/LB X 1000; MNM:MD ratio = MNM:1MD; Mortality index = MD/(MNM + MD); MMR: maternal mortality ratio = MD/LB X100.000

^a^Non-tested for influenza A(H1N1)pdm09 = includes suspected Influenza (74) + without suspected Influenza (279)

More than 55 % of patients with severe respiratory disease had three diagnostic criteria for near miss: laboratory, clinical and management, while for the remaining causes of severe maternal morbidity, around 24 % of patients only had criteria for laboratory or management diagnosis (Table 2). All procedures for management of severity were associated with a worse outcome in both groups, women with severe respiratory disease and those with severe maternal morbidity due to other causes (Table 3).

**Table 2:** Prevalence of maternal near miss criteria in women with severe respiratory disease or other causes of severe maternal morbidity

Maternal Near Miss criteria	Respiratory		Other causes		p*
	*n*	%	*N*	%	
					<0.001
Clinical only	20	6.8	93	15.0	
Laboratory only	21	7.2	151	24.4	
Management only	22	7.5	153	24.8	
Clinical + Laboratory	15	5.1	23	3.7	
Clinical + Management	41	14.0	82	13.3	
Laboratory + Management	12	4.1	30	4.9	
Clinical + Laboratory + Management	161	55.1	86	13.9	

*Adjusted for design effect of cluster sampling

**Table 3:** Estimated risk of severe maternal outcome (SMO) among women with severe respiratory disease or other causes of severe maternal morbidity according to management procedures used for severity

Procedures associated with severity	Respiratory		PR	95 % CI*	Other causes		PR	95 % CI*
	SMO	PLTC			SMO	PLTC		
Blood transfusion	146	13	**2.05**	**1.38–3.05**	397	1010	**9.78**	**7.26–13.18**
Central venous access	190	11	**2.63**	**1.71–4.06**	125	37	**13.94**	**10.30–18.88**
ICU admission	262	75	**3.84**	**1.94–7.59**	364	1414	**5.88**	**3.44–10.05**
Hospital stay >7 days	211	65	**1.97**	**1.32–2.95**	322	2270	**2.72**	**1.84–4.03**
Invasive mechanical ventilation	204	1	**3.17**	**2.08–4.81**	86	5	**15.95**	**11.85–21.48**
Use of vasoactive drug	148	0	**2.34**	**1.60–3.43**	96	0	**17.19**	**12.90–22.90**
Transfusion of ≥5U packed red blood cells	60	0	**1.83**	**1.37–2.45**	189	0	**20.70**	**16.15–26.53**
Intubation and ventilation ≥60 min not related with anesthesia	210	0	**3.35**	**2.23–5.05**	85	0	**16.86**	**12.70–22.37**
Dialysis for acute renal insufficiency	34	0	**1.75**	**1.35–2.26**	29	0	**15.35**	**11.52–20.46**
CPR	84	0	**1.93**	**1.46–2.55**	36	0	**15.52**	**11.66–20.67**

Values in bold mean they are statistically significant (*p* < 0.05)

*SMO* severe maternal outcome (maternal near miss plus maternal death); *PLTC* potentially life-threatening condition; *PR* prevalence ratio adjusted for cluster design effect; *CI* confidence interval; *ICU* intensive care unit; *CPR* cardiopulmonary resuscitation

*Adjusted for design effect of cluster sampling

Analysis of sociodemographic and obstetric characteristics (Table 4) compared cases of PLTC and SMO for both groups: with severe respiratory disease and other causes of morbidity. For the respiratory complications, there was an association to SMO and non-white color, history of diabetes, low weight and substance abuse (use of psychoactive substances, including alcohol and illicit drugs), in addition to delay in care. In contrast, the group of cases due to other causes of morbidity, low maternal age, first pregnancy, history of maternal obesity and lack of a partner were identified as having lower association to SMO, while hospitalization in a non-public institution, parity, history of caesarian section, drug abuse, complication occurrence at an earlier gestational age and mainly in the postpartum period, in addition to any type of delay in obstetric care, were associated with SMO.

**Table 4:** Estimated risk of severe maternal outcome (SMO) among women with severe respiratory disease or other causes of severe maternal morbidity according to sociodemographic and obstetric characteristics, medical history and delay in care

Variables	Respiratory		PR	95 % CI*	Other causes		PR	95 % CI*
	SMO	PLTC			SMO	PLTC		
Age (years)								
≤ 19	59	33	1.11	0.90–1.38	80	1541	**0.73**	**0.59–0.89**
20–34	176	130	1.00		401	5513	1.00	
≥ 35	57	30	1.14	0.97–1.34	137	1398	**1.32**	**1.05–1.64**
Marital status^a^								
With partner	152	100	1.00		317	3704	1.00	
Without partner	84	57	0.99	0.76–1.28	159	3466	**0.56**	**0.40–0.78**
Education^b^								
Elementary	91	62	1.00		192	2871	1.00	
> Elementary	89	62	0.99	0.77–1.28	188	3368	0.84	0.66–1.08
Skin Color^c^								
White	133	112	1.00		247	2539	1.00	
Non white	115	62	**1.20**	**1.01–1.41**	229	3702	0.66	0.43–1.01
Number of pregnancies^d^								
1	117	65	1.12	0.92–1.38	194	3599	**0.65**	**0.56–0.75**
2 or more	170	127	1.00		411	4810	1.00	
Number of childbirths^d^								
0	132	74	1.00		210	4160	1.00	
1 or more	155	118	0.89	0.73–1.08	395	4249	**1.77**	**1.55–2.02**
Previous C-sections^e^								
0	217	129	1.00		402	6363	1.00	
1 or more	61	63	0.78	0.59–1.04	184	1940	**1.46**	**1.22–1.74**
Medical history^f^								
Chronic hypertension	30	17	1.11	0.88–1.41	84	1325	0.90	0.66–1.23
Diabetes	12	3	**1.39**	**1.04–1.86**	21	173	1.69	0.94–3.07
Smoking	21	23	0.80	0.58–1.12	32	390	1.18	0.68–2.04
Obesity	39	36	0.88	0.68–1.12	66	1848	**0.46**	**0.30–0.70**
Low weight	5	0	**1.73**	**1.33–2.27**	1	21	0.70	0.09–5.30
Respiratory disease	26	31	0.76	0.48–1.20	11	166	0.96	0.62–1.46
HIV/AIDS	9	9	0.85	0.51–1.44	6	67	1.27	0.56–2.88
Substance abuse	14	4	**1.36**	**1.06–1.74**	11	71	**2.09**	**1.20–3.63**
Prenatal coverage^g^								
Public	206	134	1.00		430	5890	1.00	
Other	34	15	1.15	0.85–1.54	69	746	1.24	0.82–1.88
Prenatal adequacy^h^								
No	56	36	1.00	0.81–1.22	109	1925	**0.75**	**0.60–0.94**
Yes	212	135	1.00		476	6197	1.00	
Hospitalization coverage^i^								
Public	285	187	1.00		602	8366	1.00	
Other	6	6	0.83	0.44–1.54	16	78	**2.54**	**1.43–4.50**
Gestational age at hospital admission^j^								
< 22	36	42	0.72	0.50–1.02	47	406	**3.36**	**1.77–6.37**
22–36	161	117	0.90	0.69–1.17	297	3604	**2.47**	**1.70–3.58**
≥ 37	38	21	1.00		125	3926	1.00	
Postpartum	51	12	1.26	0.97–1.63	124	290	**9.71**	**6.20–15.20**
Delays								
Women/family members^k^	124	53	**1.42**	**1.11–1.82**	218	2902	1.14	0.89–1.44
Health service^l^	109	12	**1.85**	**1.36–2.51**	166	1089	**2.35**	**1.66–3.33**
Health professional^m^	112	9	**1.93**	**1.47–2.55**	167	1260	**2.07**	**1.41–3.04**
Any delays^n^	213	63	**2.39**	**1.60–3.56**	367	4044	**1.74**	**1.36–2.24**

*SMO* severe maternal outcome (maternal near miss plus maternal death); *PLTC* potentially life-threatening condition; *PR* prevalence ratio adjusted for cluster design effect; *CI* confidence interval; *ICU* intensive care unit

Values in bold mean that they are statistically significant (*p* < 0.05)

Missing information for: ^a^ 92 and 1424 (respiratory and other causes); ^b^ 181 and 2451; ^c^ 63 and 2353; ^d^ 6 and 56; ^e^ 15 and 181; ^f^ 43 and 1271; ^g^ 96 and 1935; ^h^ 46 and 363; ^i^ 1 and 8; ^j^ 7 and 251; ^k^ 71 and 1104; ^l^ 15 and 692; ^m^ 17 and 607; ^n^ 39 and 800 cases

*Adjusted for design effect of cluster sampling

Concerning characteristics of pregnancy and perinatal results (Table 5), the group with severe respiratory disease had a higher rate of early preterm births, between 22 and 33 weeks of gestation, low birthweight (<2500 g), Apgar < 7 at five minutes of life, stillborn and the need for hospital admission/transference of the newborn infant, compared to the group with severe maternal morbidity due to other causes. Neonatal death increased threefold in women with severe respiratory disease. A statistically significant difference was observed in the groups compared, when the mode of delivery and onset of labor were taken into consideration (*p* < 0.001). The number of women who did not undergo pregnancy resolution and remained pregnant during the severe morbid event was much higher in the respiratory disease group. Around 35 % were “still pregnant” compared to 5 % in the group with severe maternal morbidity due to other causes.

**Table 5:** Characteristics of pregnancy and perinatal results according to cause of morbidity: severe respiratory disease or other causes

Variables	Respiratory		Other causes		p*
	*n*	%	*n*	%	
Gestational age at delivery (weeks)^a^					<0.001
< 22	20	4.5	307	3.6	
22–27	23	5.2	259	3.0	
28–33	83	18.8	1341	15.6	
34–36	56	12.7	1724	20.1	
≥ 37	95	21.5	4427	51.7	
Still pregnant	164	37.2	511	6.0	
Mode of delivery^b^					<0.001
Vaginal	58	12.1	2080	23.0	
C-section	233	48.5	5921	65.5	
Abortion/ ectopic pregnancy	24	5.0	521	5.8	
Still pregnant	165	34.4	512	5.7	
Onset of labor^c^					<0.001
Spontaneous	66	14.1	2652	29.6	
Induction	33	7.1	792	8.8	
No labor	179	38.3	4477	50.0	
Abortion	24	5.1	521	5.8	
Still pregnant	165	35.3	513	5.7	
Perinatal results					
Apgar at 5 min <7^d^	38	17.7	258	3.5	<0.001
Apgar at 5 min ≥7	177	82.3	7152	96.5	
Birth weight <2500 g^e^	142	58.9	3007	39.1	<0.001
Birth weight ≥2500 g	99	41.1	4675	60.9	
Still birth^f^	36	13.6	352	4.5	<0.001
Live birth	229	86.4	7504	95.5	
Perinatal outcome^g^					<0.001
Neonatal death	15	7.0	178	2.5	
Admitted/ transferred	83	38.6	1547	21.4	
Hospital discharge	117	54.4	5506	76.1	

*Adjusted for design effect of cluster sampling

Missing information for: ^a^ 545; ^b^ 41; ^c^ 133; ^d^ 1930; ^e^ 1632; ^f^ 1434; ^g^ 2109 cases

## Discussion

Our study presents the burden of severe respiratory diseases among cases of severe maternal morbidity and results of the 2009 H1N1 influenza pandemic, considering 27 referral maternity hospitals in Brazil. Overall, the prevalence of respiratory disease was rare (5 %). Nevertheless, respiratory disease accounted for one-quarter of MNM cases and two-thirds of MD. Worse adverse outcomes occurred among cases of confirmed A(H1N1)pdm09, with an impressive MNM:MD ratio below one, meaning that there were more deaths than near miss cases in this group.

The mortality index (MI) was over 50 % in the H1N1 group, compared to 7.4 % for other causes of severe maternal morbidity. The MI is known to correlate to quality of care and when the index is above 20 %, it represents substandard care [22]. Numbers of MI over 50 % most likely reflect that poor outcomes were not only due to the severity of disease, but also to substandard care, including delays in diagnosis and management of the considered cases. Our data further confirmed that the increased risk of SMO was linked to delays in health care (delays due to women/family members, health services or health professionals).

Considering the impact of the A(H1N1)pdm09 on maternal health [10], a great effort towards prevention occurred worldwide, with strong recommendations for vaccination during pregnancy and empirical antiviral therapy, as soon as possible in case of suspected disease [10]. Brazil followed these recommendations and launched a national vaccination campaign before the winter of 2010, targeted at high-risk groups, including pregnancy. The vaccine was available in all public health facilities, at no cost for the patient and reached very high coverage (around 80 %), most likely due to the long term experience in the National Immunization Program for Children and due to the awareness about the severity of the disease, among health professionals and among the society [23]. We cannot evaluate the impact of those preventive measures in our study, since we lack information on the total number of cases and specific data on individual history of vaccination or treatment, however, from our Fig. 2, we can see on the linear traces that there is a trend towards decrease in numbers of severe cases, through time, especially after vaccination.

Clinical evaluation should determine treatment, in order to ensure timely and effective interventions. In our study, around 60 % of suspected cases of H1N1 influenza A virus were tested. In accordance with previous reports, symptoms were more severe in positive cases [24]. The majority of cases in Brazil occurred during cold weather (July, August and September), period of increased infections by respiratory viruses and influenza outbreak in the country (Brazil declared a pandemic in mid-July 2009).

Over half of the reported cases of severe respiratory disease were not due to suspected influenza infection. Acute respiratory failure was the cause, including a broad number of conditions, as follows: pulmonary edema, cardiac disease community-acquired pneumonia, aspiration, pulmonary embolism, asthma exacerbation or venous embolism. Unfortunately we do not have detailed information on each of the mentioned causes.

Nevertheless, these complications include mostly indirect causes of maternal morbidity and mortality, which represent novel or preexisting health problems unrelated to pregnancy, such as cardiac disease and asthma. Asthma is the most common medical condition that may worsen during pregnancy and it is often underdiagnosed and under-treated [9]. Direct causes of maternal morbidity and mortality can also lead to respiratory failure, such as systemic consequences of sepsis due to uterine infection and severe preeclampsia and eclampsia, complicated by pulmonary edema [25, 26].

It is very important to understand all differential diagnosis, since timely and adequate interventions can potentially improve maternal outcome. Future studies focusing on the specific differences in diagnosis and management of causes of acute respiratory failure should consider the main aspects on diagnosis and management of these conditions. Pneumonia in pregnancy and postpartum, for example, is the leading cause of fatal none obstetric infection and can be caused by bacteria, virus (at risk of secondary bacterial infection), fungus and mycobacteria and the clinical features include fever, cough, dyspnea and hypoxia [9].

Another important cause of severe complications is pulmonary edema, which can be consequence of left ventricular systolic or diastolic dysfunction, or due to the use of tocolytic agent, fluid overload, severe hypertension or severe renal disease. The clinical presentation of pulmonary edema is normally dyspnea, tachypnea, tachycardia, chest pain and diffuse crackles. There can be evidence of cardiac dysfunction, specific alterations in the electrocardiogram and radiographic abnormalities [27].

During labor or immediate postpartum, a rare and feared complication is the aspiration of gastric contents, if needed intubation for general anesthesia, due to increased intraabdominal pressure and predisposing physiological changes of pregnancy such as relaxation of the lower esophageal sphincter and delayed gastric emptying. However, in the last decades, the incidence of aspiration significantly declined, even with food intake during labor [28].

The definitions of severe respiratory complications that are usually reported can be rather confusing and sometimes difficult to incorporate [29]. Recent onset of fever and respiratory symptoms, including cough is the clinical definition of Severe Acute Respiratory Syndrome. In the setting of an epidemic, this definition is very useful to raise awareness and ensure prompt treatment, as soon as a suspected case is identified [30]. ARDS is another acronym for Acute Respiratory Distress Syndrome, a different condition that represents hypoxemic respiratory failure and bilateral radiographic opacities, without congestive heart failure. This diagnosis depends on oxygenation deficit measurements and chest imaging [31, 32].

In the current study, we couldn’t accurately establish any of the above conditions, since we did not collect data on clinical symptoms (fever, cough) neither obtained the results of those specific laboratory findings or imaging. The diagnosis of H1N1 influenza was also not standardized through all hospitals included. We understand that timing of sample collection, quality of sample and laboratory procedures are key for the accurate diagnosis and unfortunately we do not have data on details regarding these procedures.

Another limitation was the lack of data on the use of antiviral therapy or vaccination. For cases of SMO, complicated by documented organ dysfunction, ARDS would probably be the diagnosis of respiratory disease. Nevertheless, confirmation was lacking for all cases. In addition, we do not have a control group, with no underlying complication, what would be key to access risk factors. We only have data on severe maternal morbidity cases, comparing less severe (PLTC) to more severe cases (SMO).

Factors associated with SMO, included non-white color, history of diabetes, low weight and substance abuse, along with delay in care, were reported for the majority of conditions under study. Substance abuse associated with increased risk of severity in cases of respiratory disease, is in agreement with previous reports [33, 34]. Drug-related severe respiratory complications can occur, resulting from parenchymal (infectious and non-infectious pneumonitis, aspiration-related events, hemorrhage, pulmonary edema and pneumothorax), pulmonary vascular insults (endovascular infection, hemorrhage, and vasoconstriction) or airway (bronchospasm and hemorrhage) abnormalities. Diabetes was also associated with an increased risk of SMO among cases complicated by severe respiratory disease. Previous studies had demonstrated this fact, even in the Brazilian population. Diabetes is one of the main risk factors for death from H1N1 influenza A [35] virus infection. Medical history, including known factors related to worse outcomes should be highlighted and the awareness among patients and health professionals towards targeted cases could impact in the final outcome.

Pregnancy characteristics and perinatal outcomes according to the main cause of severe morbidity showed that pregnancies complicated by respiratory disease present an increased rate of preterm delivery and worse perinatal outcomes. This finding had already been demonstrated [10, 36]. Studies have shown that vaccination during the first trimester of pregnancy can improve those outcomes and decrease stillbirth rates without increasing the risk of malformations, which is a common concern among health practitioners and pregnant women [37].

## Conclusion

Severe respiratory disease, especially considering the influenza season, is one of the most serious causes of maternal near miss and death. Increased awareness of this condition, preventive vaccination during pregnancy, early diagnosis and treatment are required to improve maternal health.

## Abbreviations

SMM: severe matenal morbidity
ICU: intensive care unit
CPR: Cardiopulmonary resuscitation
PR: prevalence ratio
A(H1N1)pdm09: H1N1 influenza pandemic
ARDS: Acute Respiratory Distress Syndrome
CI: confidential interval
MD: maternal deaths
MI: mortality index
MNM: maternal near miss
PLTC: potentially life-threatening conditions
RR: respiratory rate
SMO: severe maternal outcome
WHO: World Health Organization
